# Procalcitonin Value Is an Early Prognostic Factor Related to Mortality in Admission to Pediatric Intensive Care Unit

**DOI:** 10.1155/2018/9238947

**Published:** 2018-12-24

**Authors:** Fatih Aygun

**Affiliations:** Associate Professor of Pediatrics, Istanbul University, Cerrahpasa Medical Faculty, Department of Pediatric Intensive Care Unit, Istanbul, Turkey

## Abstract

**Introduction:**

Procalcitonin (PCT) and C-reactive protein (CRP) are already known predictive markers in serious bacterial infections, and it is emphasized that these biomarkers can be used as a marker of increased mortality in critically ill patients. Herein, we aimed to evaluate the initial serum PCT and CRP levels on the outcome of patients in pediatric intensive care units (PICUs) and find out if these biomarkers can be used to predict mortality.

**Materials and Methods:**

The relationship between the initial serum PCT and CRP levels and invasive mechanical ventilation (IMV) and noninvasive mechanical ventilation (NIV) support, inotropic drug need, acute renal kidney injury (AKI), continuous renal replacement therapy (CRRT), mortality, and hospitalization period was investigated retrospectively.

**Results:**

In total, 418 suitable patients (226 males and 192 females) were included in the study. Age distributions of patients ranged from 1 month to 17 years. There was a statistically significant relationship between PCT levels in the first biochemical analysis performed during admission and MV support, inotropic drug use, mortality, ARF, hospitalization in the intensive care unit, CRRT and blood component transfusion. There was a statistically significant relationship between CRP levels and MV support, NIV, inotropic drug use, mortality, AKI, hospitalization in the intensive care unit, CRRT, and blood component transfusion.

**Conclusion:**

We suggest that the initial PCT and CRP levels during admission can be used to predict the outcome of patients in PICU.

## 1. Introduction

It is important to determine the severity and the prognostic factors of underlying disease to treat patients urgently and effectively in pediatric intensive care units (PICUs). For this reason, special scoring methods have been developed. Beside these scoring systems, some biochemical markers are also associated with the prognosis of the critically ill patients. Procalcitonin (PCT), an endogen peptide secreted in the C cells of thyroid tissue, is commonly used in the early diagnosis of sepsis and infectious diseases [1–3]. The PCT level is undetectable in plasma levels of healthy people, and the level rises in response to proinflammatory stimulus, mainly in serious bacterial infections [4]. There are various studies on adults regarding the importance of PCT in the prognosis of sepsis and critically ill patients, but there is not adequate data on children.

The level of C-reactive protein (CRP) is also very low in serum of healthy individuals like PCT but increases in various conditions like infections, malignancies, and autoimmune disorders [5]. It is already reported that the initial CRP level during admission at the intensive care unit is closely associated with the mortality and morbidity of patients [6].

Herein, with a different perspective, we aimed to evaluate the effect of initial PCT, CRP, and leucocyte levels on the outcome of critically ill patients and try to find out if these biomarkers can be used to predict mortality and morbidity of patients in PICU.

## 2. Materials and Methods

### 2.1. Study Design

The data of all patients admitted to PICU between October 2016 and January 2018 were reviewed retrospectively. Ethics committee approval was obtained from the local ethics committee (07.11.2017/775). The informed consent form was obtained from parents during admission.

We have a tertiary, multidisciplinary PICU located in a training and research hospital in Istanbul, Turkey. Our PICU provides healthcare for children from one month to 18 years; it has 12 beds, 11 ventilators, and 2 isolation rooms. Our unit employs a pediatric intensive care specialist, two assistants, and 27 nurses.

### 2.2. Patient Population and Data Collection

Patients were admitted with various causes of critical illness in the PICU. Patients discharged from the intensive care unit in less than 24 hours, patients who died on the first day after admission to the PICU, and patients with missing data were excluded from the study.

Demographic data and initial laboratory counts during admission at the intensive care unit were recorded (Table 1). The relationship between the initial PCT, CRP, and leucocyte levels and gender, age, invasive mechanical ventilation (IMV) and noninvasive mechanical ventilation (NIV) support, inotropic drug need, acute kidney injury (AKI) development, continuous renal replacement therapy (CRRT), hospitalization period, blood component transfusion and mortality was evaluated.

### 2.3. Laboratory Analysis

The initial hemogram, biochemical markers, C-reactive protein (CRP), and procalcitonin (PCT) results were recorded. For measurement of hemogram and biochemistry, peripheral blood was collected in Vacutainer tubes and analyzed by the same machines (Cell-Dyn 3700 for hemogram and Beckman Coulter for biochemistry).

### 2.4. Statistical Analysis

SPSS program (version 21.0, IBM company, SPSS Inc.) was used for statistical analysis. Continuous variables are expressed as mean ± standard deviation, and categorical variables are expressed as frequency. Pearson's chi-square and analysis of variance (ANOVA) tests were used for the comparison of categorical data between groups. The relationship between PCT, CRP, and leucocyte values was determined with receiver operating characteristic (ROC) curves. Multivariate binary logistic regression models were employed to calculate the odds ratios (ORs) with 95% confidence interval (CIs) for PCT >10 ng/mL. For all tests, *p* < 0.05 was considered to be statistically significant.

## 3. Results

### 3.1. Demographics and Prognostic Factors

Records of 447 patients were evaluated. Twenty-nine patients were not included in the study because they did not meet the study criteria; 14 patients were excluded because they did not have initial CRP, PCT, and leucocyte analysis on admission. In addition, 15 patients were excluded because they were either discharged from the intensive care unit or died in less than 24 hours. The demographic characteristics of the remaining 418 patients are shown in Table 1. Two hundred twenty-six (54.1%) patients were male and 192 (45.9%) were female. Age distributions ranged from 1 month to 17 years, and mean patient age was 3.76 ± 4.75 years. Body weight distributions ranged from 2.2 to 77 kg; the mean body weight was 11.10 ± 9.71 kg. The most frequent diagnoses of patients were respiratory disorders with 154 patients (37.2%) and the others were neurological disease, sepsis, and intoxication. The mean duration of stay in PICU was 7.56 ± 10.51 days. Invasive MV was used in 118 (32.8%) patients, and NIV was used in 201 (48.1%) patients. Acute kidney injury developed in 99 (23.7%) patients during PICU stay and 42 (10%) of these patients underwent CRRT. Thirteen of the patients (3.1%) were lost during PICU stay.

### 3.2. Relationship between Prognostic Factors and PCT, CRP, and Leucocyte Count

**Figure 1:**
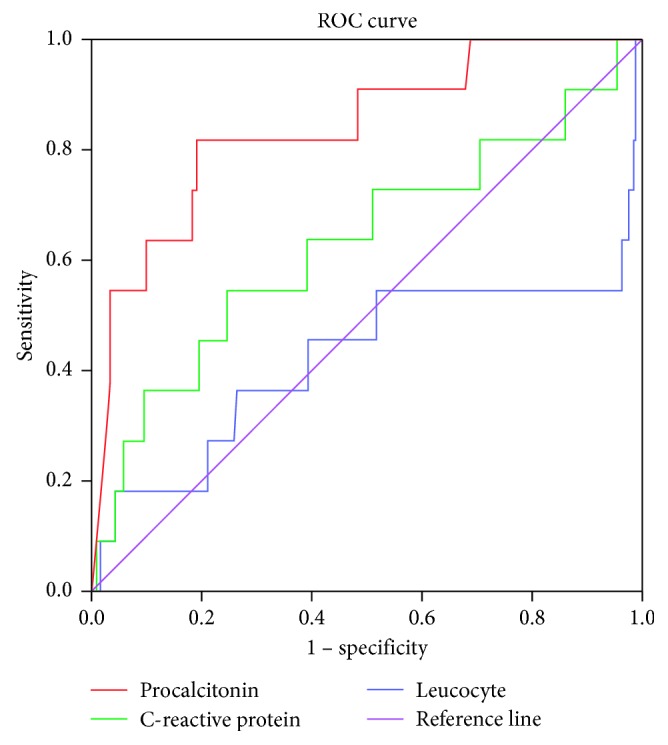
Analysis of ROC curves of correlation between mortality and biomarkers.

There was a statistically significant relationship between PCT levels in the first laboratory analysis performed during admission and MV support, inotropic drug use, mortality, ARF, hospitalization in the intensive care unit, CRRT, and blood component transfusion. *p* values were *p* ≤ 0.001, *p* ≤ 0.001, *p* ≤ 0.001, *p* ≤ 0.001, *p*=0.002, *p* ≤ 0.001, and *p* ≤ 0.001, respectively (Table 2). There was a statistically significant relationship between CRP levels during admission and MV support, NIV, inotropic drug use, mortality, ARF, hospitalization in the intensive care unit, CRRT, and blood component transfusion. *p* values were *p*=0.001, *p*=0.040, *p*=0.000, *p*=0.022, *p* ≤ 0.001, *p* ≤ 0.001, *p*=0.025, and *p* ≤ 0.001, respectively (Table 2). There was a statistically significant relationship between initial leucocyte count and NIV support and CRRT. *p* values were *p*=0.049 and *p*=0.030, respectively (Table 2). According to logistic regression analysis, the odds ratios were OR: 2.364, OR: 9.751, and OR: 2.333 for mortality, blood product transfusion, and AKI, respectively (Table 3). Analysis of ROC curves of correlation between mortality and biomarkers are showed in Table 4. PCT at a cut-off value of 6.38 ng/dl has a sensitivity of 81.8% and a specificity of 80.8% (area under curve 0.838). CRP showed 63.6% sensitivity and 61.0% specificity; leucocyte count showed 54.5% sensitivity and 51.7% specificity. Leucocyte count showed low sensitivity and specificity. The relationship graph between biomarkers and mortality is given in Figure 1.

## 4. Discussion

Infection and sepsis are among the major causes of morbidity and mortality in pediatric intensive care units. Thus, early detection of the infections in PICU is vitally important. C-reactive protein and PCT are the two most investigated and widely used biochemical markers, which are already being used in the clinical practice to evaluate the infections [7]. Procalcitonin rises more rapidly than C-reactive protein in case of infection and decline more quickly in terms of recovery [4]. Because of this feature, high PCT levels have been considered as a well diagnostic marker for septic shock [8]. Procalcitonin is also closely associated with the severity of systemic inflammation as well as bacterial infections [9]. Most of the studies in literature are focused on sepsis, and high PCT levels have been reported as a poor prognostic factor in sepsis [9, 10]. However, there are many other factors that can affect the mortality of patients in intensive care units. Acute respiratory failure requiring MV support is the major cause of hospitalization to the PICU. Mechanical ventilation does not only provide respiratory support, it also improves gas exchange and diminishes pulmonary work load. In a multicenter study performed in PICU, the frequency of MV support was 30%, and MV was associated with poor prognosis [11]. Acute kidney injury is also associated with mortality and prolonged hospitalization and considered as an independent risk factor in the bad prognosis [12, 13]. Thus, early recognition and prevention of kidney injury are of vital importance. Mechanical ventilation, inotropic drugs usage, duration of hospitalization, AKI along with CRRT, presence of sepsis, blood component transfusion, and mortality were the stated possible risk and prognostic factors, and these risk factors were considered to be indicators of our study. We evaluated the relationship between CRP, PCT, and leucocyte levels and these possible factors. There are few works in intensive care units studying on the association of these prognostic factors with PCT and CRP. Self et al. reported that patients diagnosed with community acquired pneumonia, having high PCT values (>2 ng/mL) during admission to the emergency department, are associated with increased intensive care unit need and mortality [14]. Moreover, Meng et al. performed a similar study in which increased PCT level (>10 ng/mL) during admission to the intensive care unit was strongly associated with increased mortality of the patients having different diagnosis [15]. In a prospective study concepting 81 pediatric patients, PCT values were considered as valuable marker of mortality [16]. The relationship between mortality and CRP was also evaluated in the literature. Recently, Ye et al. demonstrated a statistical relationship between CRP and mortality in 4723 pediatric intensive care patients [17]. But, the same association was not found between CRP and MV support. In the present study, mortality was significantly higher in the patients having higher CRP values, and PCT >10 mg/dl increased the mortality 2.364 times. However, we did not determine any association between mortality and leucocytosis (AUC: 0.424).

The increased PCT levels were associated with increased MV and inotropic drug usage in adults, and PCT was considered as an important biologic marker of intensive care unit need [18]. In another multicenter adult study performed by Bloos et al., PCT was associated with severity of disease in patients diagnosed with pneumonia, who needed MV support [19]. The similar relationship was found with PCT and CRP in our study; PCT and CRP levels were definitely high in patients who needed MV support. The frequency of MV need was 28.2% which was similar to the previous reports. Furthermore, blood component transfusion need and inotropic drug administration were higher in patients with PCT >10 mg/dl. Especially, the patients having PCT >10 mg/dl had significantly more blood component transfusion (OR: 9.751). In our study, ROC curves showed that PCT covered highest area under the curve (AUC: 0.838) than other parameters for mortality. In addition, ROC curves showed that PCT sensitivity (81.8%) and specificity (80.8%) was high.

There was a statistically significant association between AKI and increased PCT and CRP levels during admission. Similarly, Nie et al. suggested PCT as a predictive marker of AKI in patients having suspicion of infection [20]. Since there is not enough study on this issue, whether PCT and CRP levels increase due to AKI or AKI develops secondary to inflammation reaction itself is still unknown.

In summary, we found out that the increased PCT and CRP values during admission were considered as prognostic factors in development of AKI, MV and inotrop support, prolonged intensive care hospitalization, and mortality. Especially, PCT elevation has been shown to increase mortality.

There are some limitations of our study. Our study is a retrospective and single-centered study. There is a need for prospective and multicenter studies involving more patients. On the other hand, the fact that such a study evaluating the PCT and CRP as prognostic factor in PICU has not been done before in our country makes our study valuable.

## 5. Conclusion

The PCT and CRP values during admission to the intensive care unit can be used to predict the prognosis of critically ill patients.

## Figures and Tables

**Table 1:** Total number of patients: demographic and clinical characteristics of patients admitted to the pediatric intensive care unit from October 2016 to February 2018.

Parameter	*n* (%), mean ± standard deviation (SD)
Gender	
Male	226 (54.1%)
Female	192 (45.9%)
Diagnosis at the PICU entry	
Respiratory tract diseases	154 (36.8%)
Neurological diseases	67 (16.0%)
Septicemia	59 (14.1%)
Cardiovascular disease	38 (9.1%)
Intoxications	24 (5.7%)
Trauma	20 (4.8%)
Hematology-oncology	15 (3.6%)
Others	41 (9.8%)
Age of patients (years)	1/12–17 (3.76 ± 4.75)
Acute kidney injury	99 (23.7%)
Inotropic drug administration	95 (22.7%)
Continuous renal replacement treatment	42 (10.0%)
Mechanical ventilation application	118 (28.2%)
Length of PICU stay (days)	7.56 ± 10.51
PRISM score	14.81 ± 13.20
Mortality	13 (3.1%)
NIV application	201 (48.1%)
Number of patients administered blood product transfusion	154 (36.8%)

PICU: pediatric intensive care unit; PRISM: pediatric risk of mortality score; NIV: noninvasive mechanical ventilation.

**Table 2:** Evaluation of prognostic factors according to procalcitonin (PCT), C-reactive protein (CRP), and leucocyte values.

	PCT^1^ (ng/ml)	CRP^2^ (ml/L)	Leucocyte^3^ (mm^3^)	*p* values
MV application				≤0.001
Yes	19.76 ± 33.98	65.30 ± 85.74	12495 ± 8394	≤0.001
No	6.75 ± 18.89	39.40 ± 65.02	13553 ± 7549	0.213
NIV application				0.615
Yes	10.36 ± 24.29	54.19 ± 79.04	14028 ± 8808	0.040
No	11.77 ± 27.03	39.57 ± 64.96	12523 ± 6708	0.049
Inotropic drug administration				≤0.001
Yes	27.83 ± 37.92	78.54 ± 103.72	13484 ± 9312	≤0.001
No	5.01 ± 15.48	37.35 ± 56.90	13160 ± 7308	0.723
Mortality				≤0.001
Yes	57.41 ± 46.96	91.95 ± 118.31	12979 ± 12998	0.022
No	9.38 ± 22.97	45.27 ± 70.10	13260 ± 7603	0.898
Acute kidney injury				≤0.001
Yes	30.08 ± 38.79	74.06 ± 96.61	13565 ± 9332	≤0.001
No	4.92 ± 15.22	38.35 ± 60.83	13154 ± 7279	0.649
Length of PICU stay				0.002
<7 days	7.43 ± 21.02	35.01 ± 56.95	13344 ± 7396	≤0.001
7 days and more	16.23 ± 30.30	68.64 ± 90.87	13078 ± 8532	0.741
CRRT				≤0.001
Yes	27.75 ± 41.95	70.49 ± 102.06	10778 ± 8398	0.025
No	8.95 ± 21.99	44.05 ± 67.84	13530 ± 7696	0.030
Blood product transfusions				≤0.001
Yes	23.14 ± 34.60	73.058 ± 94.18	12548 ± 8562	≤0.001
No	2.34 ± 9.21	31.58 ± 49.58	13721 ± 7281	0.111

PCT: procalcitonin; CRP: C-reactive protein; MV: mechanical ventilation; NIV: noninvasive mechanical ventilation; CRRT: continuous renal replacement therapy.

**Table 3:** Risk factors of patients with procalcitonin >10 ng/mL^*∗*^ (logistic regression models).

Risk	*p* value	Odds ratio	95% confidence interval
Mechanical ventilation	0.281	1.532	0.705–3.332
Noninvasive mechanical ventilation	0.822	1.078	0.559–2.078
Inotropic medication	0.121	1.906	0.844–4.305
Death	0.043	2.364	0.633–5.829
Blood product transfusions	≤0.001	9.751	4.208–22.596
Acute kidney injury	0.035	2.333	1.061–5.131
Continuous renal replacement therapy	0.469	1.410	0.556–3.574

^*∗*^Multivariate logistic regression analysis.

**Table 4:** Analysis of ROC curves of correlation between mortality and biomarkers.

Parameter	Area under curve	S.E.	*p* value	95% C.I.		Cutoff value	Sensitivity (%)	Specificity (%)
				Lower bound	Upper bound			
Procalcitonin (ng/ml)	0.838	0.065	≤0.001	0.711	0.966	6.38	81.8	80.8
C-reactive protein (ml/L)	0.630	0.099	0.144	0.435	0.824	173.00	63.6	61.0
Leucocyte (mm^3^)	0.424	0.118	0.390	0.192	0.656	31.10	54.5	51.7

## Data Availability

The data used to support the findings of this study are available from the corresponding author upon request.

## Additional Points

*What This Study Adds.* (1) PCT and CRP levels can be used to evaluate the prognosis of critically ill patients. (2) PCT and CRP levels can be considered as a valuable diagnostic marker in PICU.
